# Complications of childbirth and maternal deaths in Kinshasa hospitals: testimonies from women and their families

**DOI:** 10.1186/1471-2393-11-29

**Published:** 2011-04-15

**Authors:** Eugénie Kabali, Catherine Gourbin, Vincent De Brouwere

**Affiliations:** 1 Institut Supérieur des Techniques Médicales de Kinshasa (ISTM/KIN), B.P 774, Kinshasa XI, Democratic Republic of Congo; 2 Centre de Recherche en Démographie et Sociétés, Université Catholique de Louvain, 1348 Louvain-la-Neuve, Belgium; 3 Institut de Recherche pour le Développement, UMR912, F-13500 Marseille, France and Institut National d'Administration Sanitaire, and Institut de Médecine Tropicale, Nationalestraat 155, B-2000 Antwerpen, Belgium

**Keywords:** maternal death, emergency obstetric care, childbirth, DR Congo

## Abstract

**Background:**

Maternal mortality in Kinshasa is high despite near universal availability of antenatal care and hospital delivery. Possible explanations are poor-quality care and by delays in the uptake of care. There is, however, little information on the circumstances surrounding maternal deaths. This study describes and compares the circumstances of survivors and non survivors of severe obstetric complications.

**Method:**

Semi structured interviews with 208 women who survived their obstetric complication and with the families of 110 women who died were conducted at home by three experienced nurses under the supervision of EK. All the cases were identified from twelve referral hospitals in Kinshasa after admission for a serious acute obstetric complication. Transcriptions of interviews were analysed with N-Vivo 2.0 and some categories were exported to SPSS 14.0 for further quantitative analysis.

**Results:**

Testimonies showed that despite attendance at antenatal care, some women were not aware of or minimized danger signs and did not seek appropriate care. Cost was a problem; 5 deceased and 4 surviving women tried to avoid an expensive caesarean section by delivering in a health centre, although they knew the risk. The majority of surviving mothers (for whom the length of stay was known) had the caesarean section on the day of admission while only about a third of those who died did so. Ten women died before the required caesarean section or blood transfusion could take place because they did not bring the money in time. Negligence and lack of staff competence contributed to the poor quality of care. Interviews revealed that patients and their families were aware of the problem, but often powerless to do anything about it.

**Conclusion:**

Our findings suggest that women with serious obstetric complications have a greater chance of survival in Kinshasa if they have cash, go directly to a functioning referral hospital and have some leverage when dealing with health care staff

## Background

According to estimates by the World Health Organization (WHO), there were 740 maternal deaths per 100,000 live births in the Democratic Republic of Congo (DRC) in 2005 [1]. The DRC is thus one of 17 countries with a maternal mortality ratio higher than 700 in 2005. Hogan et al. reported lower, but not statistically different, estimates of maternal mortality, around 550 in 2005 and 534 [311-856] in 2008 [2].

The most commonly suggested determinants of dismal figures like these are a low proportion of antenatal care and institutional births, both of which contribute to the facility-based approach to achieving Millennium Development Goal 5 (MDG5) [3,4]. In Kinshasa, however, the antenatal care coverage is high (96%), as is the proportion of institutional deliveries (97%) [5]. The number of doctors is in line with international standards. Indeed, the ratio of physicians per head of population is 2.4 times higher than the WHO standard of 1:10,000 inhabitants; the ratio of nurses to population is 5.6 per 5,000 inhabitants compared to WHO standards of 1:5,000 [6].

This apparent paradox of high coverage levels of institutional births with high maternal mortality level is challenging. Such a high level of maternal mortality may be explained by poor-quality care and by delays in uptake of care. Earlier studies elsewhere indicate that delays in accessing appropriate care are of paramount importance in explaining why a woman died or survived [7,8]. For Kinshasa, there is little information on the circumstances around maternal deaths. Although, part of the answer may relate to the cost and the quality of the care provided, exploring the barriers to accessing appropriate hospital care from the women's viewpoint is necessary to complete the picture.

The objective of this paper is to report the circumstances around the occurrence of complications that lead to death or near-miss, drawing on the testimonies of women who survived a serious complication, and of families of women who passed away.

## Method

Twelve hospitals were selected for the study on the basis of the largest number of deliveries and maternal deaths per year. They are spread over the 5 urban health districts of Kinshasa City. Cases were women who died in a study hospital during their pregnancy or within 42 days after delivery. The control cases were women who experienced the same complication during the same period in the same facility but who survived. We attempted to identify two controls for each case. In total 211 maternal deaths and 358 cases of serious obstetric complications were identified in the selected hospitals. Details of the general case control study are going to be submitted for publication elsewhere.

### Context

Kinshasa is the capital of the Democratic Republic of the Congo. It comprises 24 municipalities and 35 health districts. Its population has been estimated at 6 million inhabitants in 2005 [9]. Although Kinshasa has the lowest proportion of poor (41.6%) compared with the other provinces of the DR Congo (national average 71.3%), 40.0% of households just cover their needs and 40.8% are forced into debt [10]. In terms of expenditures, the poorest quartile spends on average US$ 161 per inhabitant per year (58% for food and 2.1% for health) and the least poor quartile three times more (US$ 487) [10,11]. In 2007, the total fertility rate was 3.7 children per woman in Kinshasa and the crude birth rate was 40.4‰ in urban areas [5].

### Data collection

**Figure 1:**
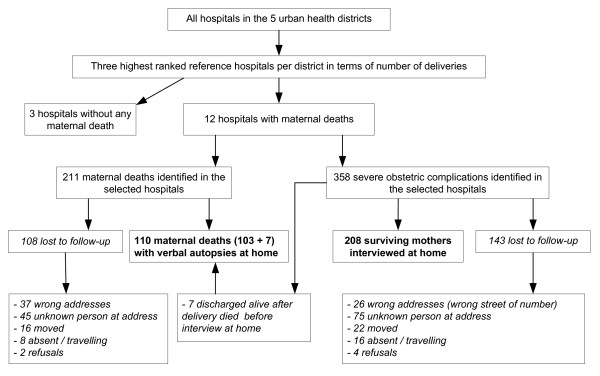
**Sample strategy**.

Interview data were collected between February and June 2005 with women admitted to the study hospitals during the year 2004. Among the 211 deceased women, it was only possible to retrieve the addresses of 103 cases. Seven additional maternal deaths were identified later among the 358 cases of women with serious obstetric complications who left the hospital alive. Relatives of the deceased were interviewed at home. Only 10% of these relatives were husbands: 66% were mothers, stepmothers, sisters or sisters in law and 24% friends or neighbours. Interviews took place on average 6.5 months after hospital discharge. Among the 358 controls, 208 surviving women were traced and interviewed (Figure 1).

A total of 318 interviews were carried out at home by three experienced female nurses (not connected with any of the selected facilities) in the local language (Lingala), using a semi-structured questionnaire (Additional file 1). The interview started by a general open question: "Could you tell me, please, what happened during your last pregnancy, in particular the course of the pregnancy, any complications or difficulties you experienced, what you have done to deal with these, and what happened in the hospital?" Case notes were immediately written in French on the questionnaire. Characteristics of the women are presented in Table 1.

**Table 1:** Characteristics of women participating in the case control study

Characteristic	Died (%)	Alive (%)	Total (%)	p
**Age**				
<20	8	9	9	0.712
20-34	66	69	68	
35 +	26	22	23	
Total (n)	(110)	(208)	(318)	
Median age	30	29	30	
**Civil status**				
Not married	19	26	24	0.092
Married	81	74	76	
Total (n)	(110)	(208)	(318)	
**Woman's level of education**				
Primary school or none	22	18	20	0.291
Secondary school or more	78	82	80	
Total (n)	(96)	(206)	(302)	
**Partner's level of education**				
Primary school or none	5	6	6	0.577
Secondary school or more	95	94	94	
Total (n)	(58)	(137)	(195)	
**Length of stay in Kinshasa**				
Resident	73	67	69	0.164
Migrant	27	33	31	
Total (n)	(98)	(204)	(302)	
**Type of care**				
Caesarean section	30	56	47	0.000
Curettage/EUA	13	16	15	-
Transfusion	31	29	30	0.049
Induction of labor	16	26	23	-
Total (n)	(110)	(208)	(318)	-
**Antenatal care (at least once)**				
Yes	71	97	88	0.000
No	29	3	12	
Total (n)	(110)	(208)	(318)	
**Type first contact facility**				
Health centre	45	24	31	
Medical health centre.	25	26	26	0.000
Hospital (reference facility)	30	50	43	
Total (n)	(110)	(208)	(318)	

This study is part of a larger case control study aiming at identifying factors associated with maternal deaths among women who experienced serious obstetric complications in Kinshasa hospitals [12].

Permission to collect data in public hospitals was given by the *Médecin Inspecteur Provincial de la Santé *(area health authority medical officer). During field work, all interviewers followed the Code of Ethics of the American Anthropological Association. The objective of the interview was explained to all the families. Confidentiality of their data and their right to refuse the interview or to stop it at any time were guaranteed.

### Data analysis

Interviews were all tape recorded and transcribed in Word. With the help of N-Vivo2 software, a content analysis of interviews has been carried out using the "three delays" model [7]. This analysis led to the formulation of themes and categories based on the most frequent answers. Data were coded according to four main themes: health care seeking behaviour, women's health behaviour, delays in seeking/obtaining care and appropriateness of health services and quality of care. A descriptive analysis of some (more frequent) codes and variables was carried out with SPSS 14.0.

## Results

The interviews with surviving women and the families of those who died from their complication showed three main problems in accessing life saving interventions: delay in seeking care when needed (1^st ^delay according to Thaddeus & Maine [7]), delay in obtaining/receiving appropriate care when in hospital (3^rd ^delay) and inadequate provision of care.

### 1. Delay in seeking care

All the women included in our study reached a hospital at some point in the development of their obstetric complication. Some women had already experienced a complication during their pregnancy (32% of the deceased women and 51% of the surviving ones). Among the 318 families interviewed, we identified 136 women (100 surviving and 36 deceased) who had experienced symptoms (danger signs) during their pregnancy. The vast majority (89%) decided to seek medical help or self-medicated (no difference between the two groups).

Some used traditional medicine when modern medicine failed: "*Pregnancy was normal until the 7^th ^month. One day, she complained of abdominal pain with fever and coughing. I accompanied her to the nearby health centre. After 4 days she became pale and her eyes yellowish. I informed my stepmother about the situation and she suggested me to find someone who could heal her with traditional medicine. However, after 4 days, the additional traditional treatment did not improve the situation, on the contrary. Then, my stepmother and I, we went to the closest maternity unit and the nurses transferred her to the referral hospital*..." (ID2026, deceased woman, 27 years, parity 0, interview with her husband).

Some women (11%) did not seek treatment, although they identified alarming symptoms during their pregnancy. They were not aware of being pregnant or neglected the danger signs. "*Pregnancy progressed with problems such as abdominal pain and vaginal bleeding until the 6^th ^month. I did not seek care because I thought these were menstruations and I did not think I was pregnant. The haemorrhage stopped spontaneously. Then, I noticed I was weak and I could not put up with the smell. I went to a polyclinic to be examined because it looked like a pregnancy. I was told it was indeed a 6 month pregnancy and I waited for one month more before attending antenatal care*." (ID1057, surviving woman, 28 years, parity 2).

Even when the complication was very obvious, some women (5 deceased and 10 surviving women) hesitated before going to the hospital. Bleeding and leaking of the amniotic fluid were not considered as danger signs, presumably because these symptoms are not painful or because the women found the quantity too small. This lack of knowledge contributed to underestimating the gravity of the complication. "*I was transferred to the intensive care unit because I bled a lot... During the pregnancy I also bled, however I found it not enough to tell the doctor about*..." (ID1154, surviving woman, 29 years, parity 2). The same behaviour occurred in case of amniotic fluid leaking; the persistence of the symptoms however contributed to the decision eventually to seek care. "*Three weeks before the expected date of delivery, I felt the leaking of liquid between my legs. However, it was not painful and I remained hopeful for one week with the leakage. As it continued, I consulted my physician who diagnosed it as amniotic fluid leakage. Because it was urgent, I accepted a third caesarean. The baby was born alive*..." (ID1199, surviving woman, 38 years, parity 4).

Lack of money is another obstacle for seeking health care, even when women are confronted with an obstetric emergency. Nine women (4 surviving and 5 deceased) tried to avoid a caesarean section because they could not afford it. They went to a health facility that clearly could not provide the care they needed or tried to deliver at home. Family often encouraged this decision. "*She had twins and the course of the pregnancy was normal except that one foetus was in the transverse lie. At the last antenatal care visit, the doctor told her: whether you feel pain or not, you must go to the hospital the day of the expected delivery date. The day came and went, but she preferred to go to church and pray with the hope of a normal delivery. When she started labour, she went to the nearby health centre... Arrived around 1:00 pm, she died soon after, waiting for money to pay for the caesarean*." (ID2049, deceased woman, 36 years, parity 2, interview with her mother). Or this other witness: "*Around 7:00 pm, I noticed a copious vaginal haemorrhage. I spoke to my husband who told me to wait, reassuring me it would stop. I changed five times my sanitary towel and I began to feel dizzy. Then he accompanied me to the nearby health centre, it was noon... the nurse said we had to go to the hospital because it was a case requiring a caesarean section. I was not convinced and went to another health centre where I was told the same. Then, I went to the referral hospital around 11:00 pm*." (ID1057, surviving woman, 28 years, parity 2).

### 2. Delay in obtaining hospital care

Money is a major obstacle to accessing emergency care. Sixty-one women (30 surviving and 31 deceased) were asked to pay before receiving any care. Ten died while family members were trying to gather the required cash (100 to 200 US$). "*She suffered from a major haemorrhage and we brought her to the medical centre and from there she was transferred to the referral hospital. The doctor told us she needed a caesarean section. Her husband left to look for money. As the bleeding continued, she died before a caesarean section was performed, with the twins still in her womb... if the caesarean had been performed, she would not have died*" (ID2074 deceased woman, 37 years, parity 6, interview with her stepmother).

Occasionally, treatment was provided before the full amount of money was handed over (n = 3, unfortunately too late for one of them). One mother had to wait more than 10 hours before the intervention, waiting for money: "... *I arrived at the referral hospital at exactly 8:00 am. I was admitted to the delivery room and after an examination, the doctor told me I had to pay 46,000CF (102US$) before the treatment. Then I was left alone the whole day. When my husband brought 30,000CF (67US$), he agreed to start the treatment. Surgery began around 7:00 pm*..." (ID1055, surviving woman, 46 years, parity 4). Another was lucky: "... *One day, I felt a little pain the whole night, then the next day around 6:00 pm I started to bleed... When I arrived at the maternity unit, I was examined and transferred to the referral hospital. We went there around 8:00 pm. After the examination, I was asked to bring enough cash for the caesarean. I spent the whole night and the next day over there, still bleeding. I was given injections to stop the bleeding. Suddenly there was heavy bleeding and the intervention was immediately performed around 5:00 pm*". (ID1061, surviving woman, 33 years parity 2).

Sometimes women's families had to resort to violence to get emergency care or to threaten the health personnel to receive care without giving an advance. "When I *arrived there, I was asked for 200US$ before admission. The soldiers who had brought me threatened the staff that they would open fire if they refused to take care of me. They were afraid and they obeyed*..." (ID1193, surviving woman, 37 years, parity 4).

The exact delay time between arrival and the appropriate intervention is known for only 89 cases (73 surviving and 16 deceased women). A proportion of 85% of surviving versus 44% of deceased women got the appropriate intervention within 24 hours, 40% versus 19% within 2 hours. The delay in getting care also affected the baby's chances of survival: 33% of newborns were stillborn or died before discharge if their mothers survived while 55% of newborns died if their mothers died.

### 3. Inappropriate hospital provision of care

We consider hospital care inadequate when there is a lack of equipment, blood, oxygen or drugs, or when the competence of the staff or their attitude is questioned.

Lack of blood has been the main factor involved in the death of 9 women. Hospital staff usually asks the family to buy blood in centres where blood is (supposedly) available. This takes time, sometimes too much time: "*they transferred the woman to the referral hospital where a caesarean section was performed around 3:00 am. The baby was stillborn. They prescribed blood but there was only one unit left in the hospital. The husband went looking for blood in another hospital, however it took too much time and the woman died around 10:00 am before her husband came back with blood*." (ID2090, deceased woman, 22 years, parity 2, interview with a woman's friend and a neighbour).

The availability of the operating theatre is also a problem. In the case of 2 surviving and 3 deceased women, the unavailability of the operating theatre was the cause of delay. "*We arrived around 9:00 pm. I was well received but had to wait my turn before the caesarean section could be performed. The operation was performed at 2:00 am*." (ID1077, surviving woman, 16 years, parity 0). And the delay is sometimes the main factor of the death: "*Around 2:00 pm, the caesarean was considered urgent. However, the operation was only performed at 11:00 pm due to the numerous operations to be performed and because everyone had to wait for her turn*". (ID2087, deceased woman, 23 years, parity 2, interview with the deceased's mother). Finally, the attitude of staff and their (perceived) competence are clearly important factors in the management of obstetric emergencies.

A few women expressed their gratitude to the health personnel (none of these had a financial problem), as they felt they were near-miss cases and would have died without the help of the health care staff. "*Two days before the labour, my whole body was swollen. When the pains started, we went to the health centre and from there to the hospital. The doctor performed a caesarean section and I was admitted in the intensive care unit of the maternity since I was in a coma. Doctors did everything possible to save my life. My baby and I, we are alive*." (ID1109, surviving woman, 19 years, parity 0). Even families of deceased women may be grateful to the hospital staff when their attitude was perceived as correct: "*There was a postpartum haemorrhage following the retained placenta. Then they removed the placenta manually and she was transfused. The doctor did everything to save her. However, death came, there was nothing we could do about it, she died the same day she delivered*". (ID2042, deceased woman, 34 years, parity 4, interview with her husband).

26 families did not really welcome the attitude of the staff. Women felt neglected: "*the pregnancy was progressing well until the 7^th ^month... Around 2:00 pm, I reached the facility where I used to attend antenatal care. I suffered from terrible backache and dizziness. My doctor was in the operating theatre. Nurses asked me to wait for him. Suddenly, I felt some liquid leaking. The nurse examined me and the cervix was only 1 cm open. Then, I started to bleed. They put me on a stretcher, waiting for the doctor. I was afraid because I had already experienced two deliveries with placenta praevia. I began to lose clots and my vision became blurred. When the doctor finished operating, the nurse did not tell him I was waiting and the doctor left. As the situation was going wrong, nurses decided to transfer me to the referral hospital*" (IUD1193, surviving woman, 37 years, parity 4).

Negligence has several forms; some of them can be really hurtful. For instance, a woman with a scar infection following a caesarean section declared she was ignored by the doctor during the daily ward rounds because the caesarean was performed by another doctor. Another woman received care only from the medical students because her family was not able to pay the full amount claimed by the doctor for the caesarean section.

When a problem of organization of care is accompanied by sheer incompetence, women's lives are clearly jeopardized: "*At the end of my pregnancy, the doctor wrote a transfer letter in advance and he requested pelvimetry. I have a congenital problem and my pelvis is too small. My doctor advised me to immediately go to a referral hospital as soon as the labour started. I went to the referral hospital for the pelvimetry but they charged 50US$ for this examination. As I had planned only 20US$ I went back home to try to gather the amount needed. The very same evening, I started labour and I went to the referral hospital. When I arrived there, I saw that the doctors were on strike. So I went to another referral hospital where I was told there was no room. Then, I went to hospital X. There, the doctor prescribed Theobald [oxytocic infusion] to stimulate the delivery. However, I showed the prescription where 'caesarean' was underlined. Another doctor came and read the prescription. He stopped the infusion and asked to prepare for an emergency caesarean... My 3.800kg baby was stillborn, I had a rupture of the uterus and they had to perform a hysterectomy. I got 3 bottles of blood. I woke up only the third day after the surgery/operation*..." (ID1176, surviving woman, 24 years, parity 0).

## Discussion

### Study limitations

We collected information about the circumstances of maternal deaths between 3 and 12 months after the death. The analysis relies on interviews of surviving women and family members of those who died. This is the first limitation of our study: respondents may seek to justify their own actions and to downplay any errors of judgment. Self-reported morbidity and verbal autopsy techniques are not very accurate methods to shed light on the medical causes of severe morbidity or death [13-16], but our study primarily sought to understand the circumstances of death and identify areas for improvement which are achievable objectives of verbal autopsies [17].

A second limitation is that the selection of women was restricted to those who reached the hospital; in a population survey those who did not reach hospital would also be represented.

The third limitation is linked to the verbal autopsy method. Informants interviewed may lack knowledge and this lack of detail may hide important features. Moreover, a comprehensive picture of the circumstances of the death could not be obtained, as we did not collect the health professional's viewpoint (notwithstanding the fact that hospital records were rarely appropriately kept).

Finally, we did not analyze individual hospital data. We cannot therefore provide an analysis by type of facility (not-for-profit or for-profit facilities) or the availability of technical equipment, both of which are known determinants of quality of care. We have tried to translate the gist of what was said by the women and their families from Lingala into French and from French into English; nevertheless in some instances we may have failed to transcribe accurately the statements of women or their families.

### Delays in obtaining care

Even when the huge majority are normal deliveries, childbirth is always a stressful event for the mother and the birth attendants. Complications requiring emergency obstetric care are usually unpredictable and obtaining prompt care is crucial [18-20]. In our study, two types of delays appeared to have contributed to the poor management of complications.

The first delay (delay in deciding to seek care on the part of the individual, the family or both) in our study was mainly due to the lack of knowledge of danger signs or to the fear of having to pay a substantial amount of money for care (the latter issue is discussed below). The lack of information about danger signs clearly reveals a problem of quality of antenatal care but also hints at a larger problem of communication. Knowledge of danger signs should be basic for all adult household members. In Tanzania, where about 98% of the women attended antenatal care at least once, only half of the women knew even one obstetric danger sign [21].

In our study, we also observed a delay in obtaining care once the woman had reached the hospital (the 3^rd ^delay). This delay was mainly due to operational factors such as shortage of medicines, blood, equipment, or to the absence of qualified staff, or their lack of competence or inappropriate attitudes, poor organization of care, or poverty, or a combination of all these factors. The 3^rd ^delay is measured by indicators such as the time elapsed between admission and the beginning of treatment, or the proportion of women treated within two hours of arrival. This statistic is used to measure the responsiveness of the hospital's emergency obstetric care [22]. This information, however, is often lacking in hospital records [23]. Nevertheless, Jahn et al. [24] showed in rural Nepal that the average time between the decision to operate and the caesarean section was 4.5 hours (40' to 11 h). In the Ivory Coast, the median duration of delay between the decision to operate and the caesarean was 2.8 h in the University Hospital and only 1 h in a referral hospital [23]. Ganatra et al. [25] showed that deceased women had a median waiting time of 12 h between their arrival and the moment they received care, longer than for the surviving ones who had the same complication (median waiting time of 5 h). In our study, the majority of the surviving women for whom the waiting time was known had their caesarean section on the day of arrival in contrast to about the third of those who died. This observation reinforces prior evidence on the delay factor in dealing with obstetric emergencies. Reducing this 3^rd ^delay is a priority that requires active management by the health care system.

### Financial barriers

In Kinshasa province in 2005, the health system consisted of 2,162 public facilities, 1,754 private for profit facilities and 164 faith-based facilities. Public and private 'for profit' hospitals ask patients to pay for their stay, medical or surgical interventions and for drugs and consumables. Drug or blood shortages however often occur in these facilities; the patient's family then has to buy the missing element from private pharmacies. Hospitals also ask for payment in advance before any intervention. Pre-payments in cash in faith-based facilities are lower than in both public and private 'for profit' hospitals (e.g. a normal delivery costs about US$ 15 compared to US$ 60 - 150 in public and private hospitals).

In the Ivory Coast Gohou et al. [23] reported a similar cause of delay in providing hospital care: "...the decision-to-delivery time was extremely long ... and this was largely determined by the time needed to obtain a complete surgical kit ..., either because the family had to pay for it in advance or because the kit lacked some essential components, which had to be bought separately...". Other studies in Bangladesh, Benin, and Morocco also showed that the lack of cash is a determinant of delay in obtaining emergency care [26-29]. A recent literature review showed the importance of financial barriers in accessing obstetric care and the social & economic consequences for the household [30,31].

According to the interviewees in our study, lack of cash was a major cause of delay in treating the patient when the complication arose. This factor was more frequently reported when the mother died. However, two families (one in a public hospital and another in a faith-based hospital) obtained treatment although the family did not pay the advance. Families attributed this compassion to the sudden severity of the complication.

In addition to the financial barrier, for many families the problem was also social disempowerment as already highlighted in the interviews of near-miss cases in Uganda [32]. In the Uganda study, disempowerment was the predominant theme among the 30 near-miss cases' interviews, both outside and inside the hospital premises when confronted with the need for rapid access to care. Very few dared to negotiate with the staff to obtain emergency care. In Tanzania, Olsen et al. [33] audited maternal deaths in two districts of the Arusha region and found a lower than average maternal death rate (382 per 100,000 live births vs 1100/100,000 as estimated by WHO). Their interpretation of this relatively low ratio was that many women experiencing complications went straight to the hospital, thus saving time and possibly their life. Moreover, women who reached the hospital were given immediate emergency obstetric services without having to secure payment first. Waiving the requirement for pre-payment also contributed to the reduction of maternal mortality.

### Inadequate services

The second factor that contributed to the delay in providing care, and sometimes the woman's death, was the chronic shortage of essential drugs and blood in hospitals. In Kinshasa, the number of facilities and qualified health workers is in theory sufficient to deal with most obstetric complications. Even for those who can afford care, there is a problem of access to drugs, blood and appropriate equipment. Timely access to blood transfusion was repeatedly identified as a problem in the limited number of studies investigating circumstances of maternal deaths in Africa [8,34-36]. In Uganda, Mbonye showed that there was no blood in some comprehensive emergency obstetric facilities; even basic equipment was often lacking [37]. In Benin, Saizonou et al. reported that sometimes the families of the near-miss women interviewed had to travel between 70 and 160 km to find blood [28].

Negligence and lack of staff competence contribute to the poor quality of care. Interviews revealed that patients and their families were aware of the problem, but often powerless to do something about it. In a study where experts rated 49 developing countries according to five features of maternal and neonatal health programmes, Congo DR was ranked 43^rd ^[38]. The weakest part of the programme was Emergency Obstetric Care (EmOC), as in the other countries. A recent review of the role of human resources and quality of obstetric care in developing countries concluded that staff shortage is a main obstacle to the provision of good quality care, that women are dissatisfied with the care they receive during childbirth and that there are not enough publications on the technical quality of EmOC [39].

## Conclusion

The analysis of perceptions of women who survived a complication and of families of women who died revealed four main problems. First, there was a major financial issue, not only for the poorest but also for the less poor who had to collect very rapidly a large sum of money before obtaining care. Second, lack of awareness of danger signs was evident, even for women who attended antenatal care. Third, staff attitude was sometimes problematic; in many cases, this reinforced the women's feeling of being mistreated by health care workers and their feeling of disempowerment. Fourth, staff attitude and the organizational capacity to provide drugs and blood promptly were also important contributing factors to the delay in providing care and to the quality of care itself.

## Competing interests

The authors declare that they have no competing interests.

## Authors' contributions

EK created the design of the study, organized the data collection, performed statistical analysis and drafted the first manuscript in French; CG participated in the design of the study, the interpretation of data and reviewed the manuscript; VDB participated in the design of the study, the interpretation of data and wrote the English version of the manuscript. All authors read and approved the final manuscript.

## Pre-publication history

The pre-publication history for this paper can be accessed here:

http://www.biomedcentral.com/1471-2393/11/29/prepub

## Supplementary Material

Additional file 1 **Questionnaire on factors associated with maternal mortality in Kinshasa**. The file is in French. The file comprises two questionnaires with closed/open questions: the first one (Fiche 4) is applied to the relatives of the deceased woman and the second one (Fiche 5) is applied to the surviving women. It consists of sections: 1. Identification of the woman; 2. socioeconomic characteristics of the family; 3. Circumstances around the complication/death; 4. Obstetric and reproductive health history; 5. History of the last pregnancy (according to the time of complication/death: before, during or after delivery); 6. Health seeking behaviour and perceived quality of care; and only for the surviving mothers, 7. Knowledge of signs of obstetric complications. Click here for file
