# Supplementary material for: Complications of childbirth and maternal deaths in Kinshasa hospitals: testimonies from women and their families
Source: BMC Pregnancy Childbirth. 2011 Apr 15;11:29. doi: 10.1186/1471-2393-11-29 (PMC3095568; doi:10.1186/1471-2393-11-29)
Supplement: Additional file 1 — Questionnaire on factors associated with maternal mortality in Kinshasa. The file is in French. The file comprises two questionnaires with closed/open questions: the first one (Fiche 4) is applied to the relatives of the deceased woman and the second one (Fiche 5) is applied to the surviving women. It consists of sections: 1. Identification of the woman; 2. socioeconomic characteristics of the family; 3. Circumstances around the complication/death; 4. Obstetric and reproductive health history; 5. History of the last pregnancy (according to the time of complication/death: before, during or after delivery); 6. Health seeking behaviour and perceived quality of care; and only for the surviving mothers, 7. Knowledge of signs of obstetric complications. [file 1471-2393-11-29-S1.DOC]

**Questionnaire d’enquête (2 fiches)**

MINISTERE DE L’ENSEIGNEMENT SUPERIEUR ET UNIVERSITAIRE/RDCONGO

ENQUETE SUR LES FACTEURS ASSOCIES A LA MORTALITE MATERNELLE A KINSHASA

**FICHE N° 4 QUESTIONNAIRE AUPRES DES PROCHES PARENTS DE LA FEMME DECEDEE (à domicile)**

N° Questionnaire  : /__/ /__/ /__/

Date de l’interview : /__/ /__/ /__//__/ /__/ /__/

Code de l’hôpital : /__/ /__/ /__/

Nom de l’enquêtrice :……………………………………………………

SECTION I : IDENTIFICATION DU REPONDANT

| 11. | Adresse | C/……………..…ZS……………………. Q/……………….Av./Rue……………N°… | |
| --- | --- | --- | --- |
| 12. | Prénom du répondant ……………………………………………………….. | | |
| 13. | Lien de parenté avec la défunte | 1= conjoint de la défunte  2= sœur/mère  3= belle sœur /belle mère  4= amie/voisine  7= autre à préciser | / _/  ………………. |
| 14. | Qui était présent auprès de la femme au moment du décès ou pendant les derniers moments de la complication ? | 1= conjoint de la défunte  2= sœur/mère  3= belle sœur /belle mère  4= amie/voisine  7= autre à préciser | / _/  ………………. |

SECTION II : CARACTERISTIQUES SOCIODEMOGRAPHIQUES DE LA DEFUNTE

| 201. | Nom de la femme décédée :……………………………………………………………. | | |
| --- | --- | --- | --- |
| 202. | Date du décès |  | /_/_ //_/_//_/_/ |
| 203. | Date de naissance |  | /_/_//_/_//_/_/ |
| 204. | Age révolu (années) |  | /_/_/ |
| 205. | Province d’origine | 01 = Bas-congo  02 = Bandundu  03 = Equateur  04 = Province orientale  05 = Nord-Kivu  06 = Sud-Kivu  08 = Maniema  10 = Kasai oriental  11 = Kasai occidental  12 = Katanga  13 = Kinshasa  14 = hors RDC  9 = nsp | /_/_/ |
| 206. | Durée de résidence à Kinshasa | /_/_ / mois /_/_/ années 00 = native 99= nsp | |
| 207 | Niveau d’instruction | 1= sans instruction  2= primaire  3= secondaire  4= supérieur et+  9= nsp | /__/ |
| 208 | Profession/activités rémunératrices | 1= ménagère/sans emploi  2= petit commerce, couture,…  5= agent de la fonction publique  6= cadre de la fonction publique  8= médecin, avocat, ingénieurs,  9= nsp | /__//__/ |
| 209 | Situation matrimoniale | 1= célibataire → 212  2= mariée  3= veuve/séparée/divorcée→212  7= autre……………………..  9= nsp | /__/ |
| 210 | Niveau d’instruction  du conjoint | 1= sans instruction  2= primaire  3= secondaire  4= supérieur et+  9= nsp | /__/ |
| 211 | Profession/activités rémunératrices du conjoint | 1= sans emploi/ retraité  2= petit commerce, couture,…  5= agent de la fonction publique  6= cadre de la fonction publique  8=médecin, avocat, ingénieurs,  9= nsp | /__/ |
| 212 | Type d’habitat | 1= villa  2= maison individuelle  3= Immeuble/ONL  4= construction précaire  7= autre(à préciser) | /__/ |
| 213 | Mode d’approvisionnement en eau | 1= eau courante dans la parcelle  2= eau courante en dehors de la parcelle  3= eau de puit  7= autre à préciser | /__/  ………………. |
| 214 | Nature toiture | 1= tuile  2= tôle  3= pisée  7= autre à préciser | /__/  ………………. |
| 215 | Nature mur | 1= dur  2= pisée  7= autre à préciser | /__/  ………………. |
| 216 | Nature sol | 1= terre battue  2= ciment  3= carrelage  7= autre à préciser | /__/  ………………. |

SECTION III : CIRCONSTANCES DU DECES

| Pouvez-vous me parler des circonstances autour de sa dernière grossesse qui ont conduit au décès (évolution de la grossesse, complication, décès, en particulier au sujet des complications ou difficultés : ce que vous avez fait, ce qu’on a fait, ce que vous auriez pu faire, ce qu’on aurait du faire à l’hôpital…………..) |
| --- |
| ………………………………………………………………………………………………………………………………………………………………………………………………………………………………………………………………………………………………………………………………………………………………………  ………………………………………………………………………………………………………………………………………………………………………………………………………………………………………………………………………………………………………………………………………………………………………  ………………………………………………………………………………………………………………………………………………………………………………………………………………………………………………………………………………………………………………………………………………………………………  ………………………………………………………………………………………………………………………………………………………………………………………………………………………………………………………………………………………………………………………………………………………………………  ………………………………………………………………………………………………………………………………………………………………………………………………………………………………………………………………………………………………………………………………………………………………………  ………………………………………………………………………………………………………………………………………………………………………………………………………………………………………………………………………………………………………………………………………………………………………  ………………………………………………………………………………………………………………………………………………………………………………………………………………………………………………………………………………………………………………………………………………………………………  ………………………………………………………………………………………………………………………………………………………………………………………………………………………………………………………………………………………………………………………………………………………………………  ………………………………………………………………………………………………………………………………………………………………………………………………………………………………………………………………………………………………………………………………………………………………………  ………………………………………………………………………………………………………………………………………………………………………………………………………………………………………………………………………………………………………………………………………………………………………  ………………………………………………………………………………………………………………………………………………………………………………………………………………………………………………………………………………………………………………………………………………………………………  ………………………………………………………………………………………………………………………  ………………………………………………………………………………………………………………………………………………………………………………………………………………………………………………………………………………………………………………………………………………………………………  ………………………………………………………………………………………………………………………  ………………………………………………………………………………………………………………………………………………………………………………………………………………………………………………………………………………………………………………………………………………………………………  ………………………………………………………………………………………………………………………………………………………………………………………………………………………………………………………………………………………………………………………………………………………………………  …………………………………………………………………………………………………………………………………………………………………………… |

# Si nécessaire continuer sur une autre page SVP!!!

# SECTION IV : ANTECEDENTS MEDICAUX ET HISTOIRE GENESIQUE

| 41. | La défunte souffrait-elle d’une maladie chronique | 1= oui  2= non → 43  9= nsp → 43 | /__/ | |
| --- | --- | --- | --- | --- |
| 42. | Si oui, laquelle | 1= HTA  2= Anémie  3= asthme  4= diabète  5= TBC  6= SIDA  7= autre  9= nsp | /__/ | |
| 43. | Nombre de grossesses antérieures | /__//__/ 99= nsp | | |
|  | Nombre d’enfants nés vivants | /__//__/ 99= nsp | | |
| 44. | Nombre d’avortements /morts-nés antérieurs | /__//__/ 99= nsp | | |
| 45. | Nombre d’enfants décédés | /__//__/ 99= nsp | | |
| 46. | Nombre d’enfants encore en vie | /__//__/ 99= nsp | | |
| 47. | Nombre de césarienne | /__//__/ 99= nsp | | |
| 48. | Autres antécédents obstétricaux | | | |
| 49. | La femme est-elle décédée | 1= pendant la grossesse →section 5A  2= pendant le travail →section 5B  3= après l’accouchement →section 5C | | /__/ |

**SECTION V. GROSSESSE AYANT ENTRAINE LE DECES**

## A. FEMMES DECEDEES PENDANT LA GROSSESSE MAIS AVANT LE DEBUT DU TRAVAIL

| 501. | Temps entre le début de la complication et la décision d’aller à l’hôpital | /_/_ / min /_/_/h /_/_/j 99) nsp | /__/__/ |
| --- | --- | --- | --- |
| 502. | Temps entre le début de la complication et la décision d’aller à l’hôpital |  |  |
| 503. | Au cours de cette grossesse qui a entraîné le décès, la femme :  A-t-elle eu d’épisodes de saignement ? | 1= oui  2= non  9= nsp | /__/ |
| 504. | Le saignement était-il | 1= peu abondant (souiller une bande hygiénique par jour)  2= abondant (souiller 4 bandes hygiéniques par jour)  3= très abondant (souiller plusieurs bandes hygiéniques par jour) | /__/ |
| 505. | Qu’avait-on fait pour l’arrêter ? | | |
| 506. | A-t-elle eu une pâleur | 1= oui  2= non  9= nsp | /__/ |
| 507. | A-t-elle eu des Frissons | 1= oui  2= non  9= nsp | /__/ |
| 508. | A-t-elle eu des douleurs abdominales | 1= oui  2= non  9= nsp | /__/ |
| 509. | A-t-elle eu des douleurs pelviennes | 1= oui  2= non  9= nsp | /__/ |
| 510. | A-t-elle eu des Convulsions | 1= oui  2= non  9= nsp | /__/ |
| 511. | A-t-elle eu des oedèmes exagérés (visage/ jambes gonflées) | 1= oui  2= non  9= nsp | /__/ |
| 512. | Avait-elle eu fièvre élevée (> 24heures) | 1= oui  2= non  9= nsp |  |
| 513. | Autres symptômes/pathologies observées ou dont la femme vous avait parlé | | |

**Maintenant passer à la section VI**

## B. FEMMES DECEDEES PENDANT LE TRAVAIL MAIS AVANT L’EXPULSION

| 501. | Temps entre le début du travail et la décision d’aller à l’hôpital | /_/_ / min /_/_/h /_/_/j 99) nsp | /__/__/ |
| --- | --- | --- | --- |
| 502. | Temps entre le début du travail et le décès | /_/_ / min /_/_/ h /_/_/ j 99) nsp | /__/__/ |
| 503. | La poche des eaux était-elle rompue avant le début du travail | 1= oui  2= non  9= nsp | /__/ |
| 504. | Qui a surveillé la femme durant le travail | 1= médecin  2= accoucheuse/infirmière  3= aide accoucheuse  7= autre (préciser)  9= nsp | /__/ |
| 505. | La femme avait-elle pris des médicaments durant le travail | 1= oui  2= non →606  9= nsp →606 | /__/ |
| 506. | Si oui, nom, dose et nombre de prise du médicament :…………………………………………  …………………………………………………………………………………………………………… | | |
| 507. | Voie d’administration | 1= IV  2= IM  3= Perfusion  4= orale  5=lavement 9)nsp | /__/ |
| 508. | A-t-elle eu des saignements | 1= oui  2= non  9= nsp | /__/ |
| 509. | Le saignement était-il | 1= peu abondant (souiller une bande hygiénique par jour)  2= abondant (souiller 4 bandes hygiéniques par jour)  3= très abondant (souiller plusieurs bandes hygiéniques par jour) | /__/ |
| 510. | Qu’avait-on fait pour l’arrêter ? | | |
| 511. | A-t-elle eu des douleurs abdominales | 1= oui  2= non  9= nsp | /__/ |
| 512. | A-t-elle eu des douleurs pelviennes | 1= oui  2= non  9= nsp | /__/ |
| 513. | A-t-elle eu des Convulsions | 1= oui  2= non  9= nsp | /__/ |
| 514. | A-t-elle eu des oedèmes exagérés (visage/ jambes gonflées) | 1= oui  2= non  9= nsp | /__/ |
| 515. | Avait-elle eu fièvre élevée (> 24heures) | 1= oui  2= non  9= nsp |  |
| 516. | Autres symptômes/pathologies observées ou dont la femme vous avait parlé | | |

**Maintenant passer à la section VI**

**C. FEMMES DECEDEES APRES EXPULSION (ACCOUCHEMENT)**

| 501. | Préciser le lieu d’accouchement | 1= domicile  2= en route vers l’hôpital  3= Centre de Santé  4= hôpital  9= nsp | /__/ |
| --- | --- | --- | --- |
| 502. | Qualité de la personne qui a assisté à l’accouchement | 1=médecin  2=accoucheuse/infirmière  3=aide accoucheuse  4=matrones  7=autres 9= nsp | /__/  ………………. |
| 503. | Temps entre le début du travail et la décision d’aller à l’hôpital | /_/_ / min /_/_/h /_/_/j 99) nsp | /__/__/ |
| 504. | Temps l’accouchement (expulsion) et le décès | /_/_ / min /_/_/h /_/_/j 99) nsp | /__/__/ |
| 506. | A-t-elle eu des saignements | 1= oui  2= non  9= nsp | /__/__/ |
| 507. | Le saignement était-il | 1= peu abondant (souiller une bande hygiénique par jour)  2= abondant (souiller 4 bandes hygiéniques par jour)  3= très abondant (souiller plusieurs bandes hygiéniques par jour) | /__/ |
| 508. | Qu’avait-on fait pour l’arrêter ? | | |
| 509. | A-t-elle eu des douleurs abdominales | 1= oui  2= non  9= nsp | /__/ |
| 510. | A-t-elle eu des douleurs pelviennes | 1= oui  2= non  9= nsp | /__/ |
| 511. | A-t-elle eu des Convulsions | 1= oui  2= non  9= nsp | /__/ |
| 512. | Avait-elle eu une fièvre élevée  (> 24heures) | 1= oui  2= non  9= nsp | /__/ |
| 513 | Autres symptômes/pathologies observées ou dont la femme vous avait parlé | | |
| 514. | Statut de l’enfant à l’accouchement | 1= vivant  2= mort-né  9= nsp | /__/ |
| 515. | Statut actuel de l’enfant | 1= vivant  2= mort-né  9= nsp | /__/ |
| 516. | Délai entre décès mère et décès enfant | /__//__/ J /__//__/ S /__//__/ M |  |

**SECTION VI : COMPORTEMENT SANITAIRE ET QUALITE DES SOINS**

| 601 | Préciser l’itinéraire thérapeutique suivi par la femme entre le début du travail et le décès | | |
| --- | --- | --- | --- |
| 602 | La femme avait-elle été aux CPN au cours de la dernière grossesse | 1= oui  2= non → 605  9= nsp → 605 | /__/ |
| 603 | Avait-elle commencé les CPN | 1= 1^er^ trimestre  2=2^ème^ trimestre  3=3^ème^ trimestre  9=nsp | /__/ |
| 604 | Où avait-elle suivi les CPN ? | 1= CS  2= CM/Polyclinique  3= hôpital  9= nsp | /__/ |
| 605 | Qui l’avait examiné pendant les CPN ? | 1= médecin  2= accoucheuse/infirmière  3= aide accoucheuse  4= matrone  9= nsp | /__/ |
| 606 | La femme avait-elle prévu d’accoucher dans une formation sanitaire? | 1= oui  2= non  9= nsp | /__/ |
| 607 | Avait-elle accouché dans la formation prévue ? | 1= oui → 608  2= non  9= nsp → 608 | /__/ |
| 608 | Si non pourquoi ? | | |
| 609 | Etait-elle directement prise charge dès son arrivée à l’hôpital ? | 1= oui → 710  2= non  9= nsp | /__/ |
| 610 | Etait-elle restée combien de temps à l’hôpital avant d’être prise en charge | /__//__/Min/__//__/ Hrs; 99 = nsp | /__/ |
| 611 | Selon vous, qu’est-ce qu’on aurait pu faire au niveau de l’hôpital pour éviter le décès | | |
| 612 | Avez-vous d’autres commentaires sur le décès que nous n’avons pas évoqué ci-dessus ? | | |

**Vérifier que tout est rempli, REMERCIER L’ENQUETE et arrêter l’interview**

**Section finale : Enquêtrice, prière de noter :**

**1) les problèmes pour trouver le meilleur répondant**

1. **votre commentaire sur le déroulement de l’interview**
2. **votre satisfaction sur la coopération de l’enquêté: 1= TB ; 2= B ; 3= M**

MINISTERE DE L’ENSEIGNEMENT SUPERIEUR ET UNIVERSITAIRE/RDCONGO

**ENQUETE SUR LES FACTEURS ASSOCIES A LA MORTALITE MATERNELLE A KINSHASA**

**FICHE N°5 QUESTIONNAIRE AUPRES DES FEMMES SURVIVANTES  (à domicile)**

N° questionnaire /__/ /__/ /__/

Date de l’interview/__/ /__/ /__//__/ /__/ /__/

Code: /__/ /__/

Nom de l’enquêtrice : ……………………………………………………

# SECTION I : IDENTIFICATION DU REPONDANT

| 11. | Adresse | | | C/……………..…ZS……………………. Q/……………….Av./Rue……………N°… | |
| --- | --- | --- | --- | --- | --- |
| 12. | Prénom du répondant ………………………………………………………………………………… | | | | |
| 13. | Lien de parenté avec la femme survivante | | 1= survivante  2= conjoint de la femme  3= sœur/mère  4= belle sœur /belle mère  5= amie/voisine  7= autre à préciser  9= nsp | | / _/  ………………. |
| 14. | La femme a migré pour | | | | |
| 15. | La femme est décédée le / _// _// _// _// _// _/ | | | | |
| 16 | Qui avait accompagné la femme lors du dernier accouchement ? | 1= conjoint  2= sœur/mère  3= belle sœur /belle mère  4= amie/voisine  5= personne  7= autre à préciser  9= nsp | | | / _/  ………………. |

# SECTION II : CARACTERISTIQUES SOCIO DEMOGRAPHIQUES DE LA SURVIVANTE

|  | 201. | Nom de la femme :……………………………………………………………. | | |
| --- | --- | --- | --- | --- |
|  | 202. | Date de naissance |  | /_/_ //_/_//_/_/ |
|  | 203. | Age révolu (années) |  | /_/_/ |
|  | 204. | Date d’accouchement |  | /_/_//_/_//_/_/ |
|  | 205. | Province d’origine | 01 = Bas-congo  02 = Bandundu  03 = Equateur  04 = Province orientale  05 = Nord-Kivu  06 = Sud-Kivu  08 = Maniema  10 = Kasai oriental  11 = Kasai occidental  12 = Katanga  13 = Kinshasa  14 = hors RDC  9 = nsp | /_/_/ |
|  | 206. | Durée de résidence à Kinshasa | /_/_ / mois /_/_/ années 00 = native 99= nsp | |
|  | 207 | Niveau d’instruction | 1= sans instruction  2= primaire  3= secondaire  4= supérieur et+  9= nsp | /__/ |
|  | 208 | Profession/activités rémunératrices | 1= ménagère/sans emploi  2= petit commerce, couture,…  5= agent de la fonction publique  6= cadre de la fonction publique  8= médecin, avocat, ingénieurs,  9= nsp | /__//__/ |
|  | 209 | Situation matrimoniale | 1= célibataire → 212  2= mariée  3= veuve/séparée/divorcée→212  7= autre……………………..  9= nsp | /__/ |
|  | 210 | Niveau d’instruction  du conjoint | 1= sans instruction  2= primaire  3= secondaire  4= supérieur et+  9= nsp | /__/ |
|  | 211 | Profession/activités rémunératrices du conjoint | 1= sans emploi/ retraité  2= petit commerce, couture,…  5= agent de la fonction publique  6= cadre de la fonction publique  8=médecin, avocat, ingénieurs,  9= nsp | /__/ |
|  | 212 | Type d’habitat | 1= villa  2= maison individuelle  3= Immeuble/ONL  4= construction précaire  7= autre(à préciser) | /__/ |
|  | 213 | Mode d’approvisionnement en eau | 1= eau courante dans la parcelle  2= eau courante en dehors de la parcelle  3= eau de puit  7= autre à préciser | /__/  ………………. |
|  | 214 | Nature toiture | 1= tuile  2= tôle  3= pisée  7= autre à préciser | /__/  ………………. |
|  | 215 | Nature mur | 1= dur  2= pisée  7= autre à préciser | /__/  ………………. |
|  | 216 | Nature sol | 1= terre battue  2= ciment  3= carrelage  7= autre à préciser | /__/  ………………. |
| **SECTION III.** **CIRCONSTANCES DE LA COMPLICATION**  Pouvez-vous me parler en détail, s’il vous plaît, des événements qui ont entouré la dernière grossesse (en particulier au sujet des complications ou difficultés : évolution de la grossesse, ce que vous avez fait, ce qu’on a fait, ce que vous auriez pu faire, ce qu’on aurait du faire à l’hôpital…………..)  ………………………………………………………………………………………………………………………………………………………………………………………………………………………………………………………………………………………………………………………………………………………………………  ………………………………………………………………………………………………………………………………………………………………………………………………………………………………………………………………………………………………………………………………………………………………………  ………………………………………………………………………………………………………………………………………………………………………………………………………………………………………………………………………………………………………………………………………………………………………  ………………………………………………………………………………………………………………………………………………………………………………………………………………………………………………………………………………………………………………………………………………………………………  ………………………………………………………………………………………………………………………………………………………………………………………………………………………………………………………………………………………………………………………………………………………………………  ………………………………………………………………………………………………………………………………………………………………………………………………………………………………………………………………………………………………………………………………………………………………………  ………………………………………………………………………………………………………………………………………………………………………………………………………………………………………………………………………………………………………………………………………………………………………  ………………………………………………………………………………………………………………………………………………………………………………………………………………………………………………………………………………………………………………………………………………………………………  ………………………………………………………………………………………………………………………………………………………………………………………………………………………………………………………………………………………………………………………………………………………………………  ………………………………………………………………………………………………………………………………………………………………………………………………………………………………………………………………………………………………………………………………………………………………………  ………………………………………………………………………………………………………………………………………………………………………………………………………………………………………………………………………………………………………………………………………………………………………  ………………………………………………………………………………………………………………………  ………………………………………………………………………………………………………………………………………………………………………………………………………………………………………………………………………………………………………………………………………………………………………  ………………………………………………………………………………………………………………………………………………………………………………………………………………………………………………………………………………………………………………………………………………………………………  ………………………………………………………………………………………………………………………  ………………………………………………………………………………………………………………………………………………………………………………………………………  ……………………………………………………………………………………………………………………… | | | | |

# Si nécessaire, continuer sur une autre page SVP !!!

# SECTION IV : ANTECEDENTS MEDICAUX ET HISTOIRE GENESIQUE

| 41 | Souffrez-vous d’une maladie chronique | 1= oui  2= non →303  9=nsp →303 | /__/ |
| --- | --- | --- | --- |
| 42 | Si oui, laquelle | 1= HTA  2= Anémie  3= asthme  4= diabète  5= TBC  6= Sida  7= autre  9= nsp | /__/ |
| 43 | Nombre de grossesses antérieures | /__//__/ 99= nsp | |
| 44 | Nombre d’enfants nés vivants | /__//__/ 99= nsp | |
| 45 | Nombre d’avortements /morts-nés antérieurs | /__//__/ 99= nsp | |
| 46 | Nombre d’enfants décédés | /__//__/ 99= nsp | |
| 47 | Nombre d’enfants nés vivants | /__//__/ 99= nsp | |
| 48 | Nombre de césarienne | /__//__/ 99= nsp | |
| 49 | Autres antécédents obstétricaux | | |

**SECTION V. GROSSESSE AYANT ENTRAINE LA COMPLICATION**

**A. FEMMES AYANT CONNU LA COMPLICATION PENDANT LE TRAVAIL AVANT L’EXPULSION**

| A. 501. | Temps entre le début du travail et la décision d’aller à l’hôpital | /_/_ / min /_/_/h /_/_/j 99) nsp | /__/__/ |
| --- | --- | --- | --- |
| A. 502. | Temps entre le début du travail et la survenue de la complication | /_/_ / min /_/_/ h /_/_/ j 99) nsp | /__/__/ |
| A. 503. | La poche des eaux était-elle rompue avant le début du travail | 1= oui  2= non  9= nsp | /__/ |
| A. 504. | Qui a surveillé la femme durant le travail | 1= médecin  2= accoucheuse/infirmière  3= aide accoucheuse  7= autre (préciser)  9= nsp | /__/ |
| A. 505. | La femme avait-elle pris des médicaments durant le travail | 1= oui  2= non →506  9= nsp →506 | /__/ |
| A. 506. | Si oui, nom, dose et nombre de prise du médicament :…………………………………………  …………………………………………………………………………………………………………… | | |
| A. 507. | Voie d’administration | 1= IV  2= IM  3= Perfusion  4= orale  5=lavement 9)nsp | /__/ |
| A. 508. | Avez-vous eu des saignements | 1= oui  2= non  9= nsp | /__/ |
| A. 509. | Le saignement était-il | 1= peu abondant (souiller une bande hygiénique par jour)  2= abondant (souiller 4 bandes hygiéniques par jour)  3= très abondant (souiller plusieurs bandes hygiéniques par jour) | /__/ |
| A. 510. | Qu’avait-on fait pour l’arrêter ? | | |
| A. 511. | Avez-vous eu des douleurs abdominales | 1= oui  2= non  9= nsp | /__/ |
| A. 512. | Avez-vous eu des douleurs pelviennes | 1= oui  2= non  9= nsp | /__/ |
| A. 513. | Avez-vous eu des Convulsions | 1= oui  2= non  9= nsp | /__/ |
| A. 514. | Avez-vous eu des oedèmes exagérés (visage/ jambes gonflées) | 1= oui  2= non  9= nsp | /__/ |
| A. 515. | Avez-vous eu une fièvre élevée  (> 24heures) | 1= oui  2= non  9= nsp |  |
| A. 516. | Autres symptômes/pathologies | | |

**Maintenant passer à la section VI**

**B. FEMMES DECEDEES APRES EXPULSION (ACCOUCHEMENT)**

| B.501. | Temps entre le début du travail et la décision d’aller à l’hôpital | /_/_ / min /_/_/h /_/_/j 99) nsp | /__/__/ |
| --- | --- | --- | --- |
| B.502. | Temps entre l’accouchement (expulsion) et la survenue de la complication | /_/_ / min /_/_/h /_/_/j 99) nsp | /__/__/ |
| B.503. | Avez-vous eu des saignements | 1= oui  2= non  9= nsp | /__/__/ |
| B.504. | Le saignement était-il | 1= peu abondant (souiller une bande hygiénique par jour)  2= abondant (souiller 4 bandes hygiéniques par jour)  3= très abondant (souiller plusieurs bandes hygiéniques par jour) | /__/ |
| B.506. | Qu’avait-on fait pour l’arrêter ? | | |
| B.507. | Avez-vous eu des douleurs abdominales | 1= oui  2= non  9= nsp | /__/ |
| B.508. | Avez-vous eu des douleurs pelviennes | 1= oui  2= non  9= nsp | /__/ |
| B.509. | Avez-vous eu des Convulsions | 1= oui  2= non  9= nsp | /__/ |
| B.510. | Avez-vous eu une fièvre élevée  (> 24heures) | 1= oui  2= non  9= nsp | /__/ |
| B.11. | Autres symptômes/pathologies observées ou dont la femme vous avait parlé | | |
| B.512. | Préciser le lieu d’accouchement | 1= domicile  2= en route vers l’hôpital  3= Centre de Santé  4= hôpital  9= nsp | /__/ |
| B.513 | Qualité de la personne qui a assisté à l’accouchement | 1=médecin  2=accoucheuse/infirmière  3=aide accoucheuse  4=matrones  7=autres 9= nsp | /__/  ………………. |
| B.514. | Statut de l’enfant à l’accouchement | 1= vivant  2= mort-né  9= nsp | /__/ |
| B.515. | Statut actuel de l’enfant | 1= vivant  2= mort-né  9= nsp | /__/ |

**SECTION VI : COMPORTEMENT SANITAIRE ET QUALITE DES SOINS**

| 601 | Préciser l’itinéraire thérapeutique que vous avez suivi entre le début du travail et la survenue de la complication | | | | | |
| --- | --- | --- | --- | --- | --- | --- |
| 602 | Avez-vous été aux CPN au cours de la dernière grossesse | | | 1= oui  2= non → 605  9= nsp → 605 | /__/ | |
| 603 | Avez-vous commencé les CPN | | | 1= 1^er^ trimestre  2=2^ème^ trimestre  3=3^ème^ trimestre  9=nsp | /__/ | |
| 604 | Où avez-vous suivi les CPN ? | | | 1= CS  2= CM/Polyclinique  3= hôpital  9= nsp | /__/ | |
| 605 | Qui vous avait examiné pendant les CPN ? | | | 1= médecin  2= accoucheuse/infirmière  3= aide accoucheuse  4= matrone  9= nsp | /__/ | |
| 606 | Avez-vous prévu d’accoucher dans une formation sanitaire? | | | 1= oui  2= non  9= nsp | /__/ | |
| 607 | Avez-vous accouché dans la formation prévue ? | | | 1= oui → 609  2= non  9= nsp → 609 | /__/ | |
| 608 | Si non pourquoi ? | | | | | |
| 609 | Avez-vous été à une consultation post-natale ? | | | 1= oui  2= non  9= nsp | /__/ | |
| 610 | Quand vous êtes arrivé à l’hôpital pour l’accouchement avez-vous attendu avant d’être reçu | | 1=oui  2=non →703  9=nsp →703 | | | /__/ |
| 611 | Aviez-vous attendu pendant combien de temps?  /__//__/ min /__//__/heures ; 99= nsp | | | | | /__//__/ |
| 612 | Aviez-vous senti votre intimité respectée au dernier accouchement | | 1=oui  2=non  9=nsp | | | /__/ |
| 613 | En quoi avez-vous senti cela ? | | | | | |
| 614 | Qu’est-ce qui vous a marqué positivement en ce qui concerne le personnel ? (plusieurs réponses possibles) | | 1= bon comportement personnel  2= disponibilité médicaments  3= ne pas devoir attendre  4= traitement effectif (guérit)  5= répondre aux questions  6= propreté  7=autre  8= amitié et communication | | | /__/ |
| 615 | Qu’est-ce qui vous a marqué négativement en ce qui concerne le personnel? (plusieurs réponses possibles) | | 1= mauvais comportement personnel  2= non disponibilité médicaments  3= lenteur du traitement  4= traitement ne guérit pas  5= ne pas répondre aux questions  6= manque de propreté  7= autre  8= manque d’amitié et communication | | | /__/ |
| 616 | Le médecin vous avait-il expliqué les problèmes que vous aviez? | | 1=oui  2=non  9=nsp | | | /__/ |
| 617 | Le médecin vous avait-il expliqué le traitement qu’il allait vous administrer ? | | 1=oui  2=non  9=nsp | | | /__/ |
| 618 | Pensez-vous que c’était nécessaire | | 1=oui  2=non  9=nsp | | |  |
| 619 | Pourquoi ? | | | | | |
| 620 | Quelles sont les choses que vous avez eu à acheter en dehors de l’hôpital | | 1= tous les médicaments  2= certains médicaments  3=sang transfusion  4= seringues et aiguilles  5= aucun (médicament, matériel)  6= tout (médicament, matériel)  7= autres | | | /__/ |
| 621 | Quel a été le coût total (approx.) du traitement reçu à l’hôpital ?:  /__//__//__//__//__//__/FC ; 99=nsp | | | | |  |
| 622 | Comment avez-vous réglé la facture | 1= payez totalité cash  2= cash et gage  3=totalité gage  4= cash et bienfaiteurs  5= dons/bienfaiteurs  7= autres moyens  9=nsp | | | | /__/ |
| 623 | Les autres membres de la famille avaient-ils contribué aux dépenses liées à l’accouchement ? | 1=oui  2=non  9=nsp | | | | /__/ |
| 624 | Avez vous d’autres commentaires à faire sur l’accouchement que nous n’avons pas abordé ci-dessus ? | 1=oui  2=non  9=nsp | | | | /__/ |
| 624 | Lesquels | | | | |  |

SECTION VII : CONNAISSANCE DES COMPLICATIONS OBSTETRICALES

| 701 | Selon vous, la grossesse constitue-t-elle un risque de décès pour la femme ? | 1= oui  2= non 9= nsp | /__/ |
| --- | --- | --- | --- |
| 702 | Pourquoi | | |
| 703 | Que faut-il faire en cas d’une complication liée à la grossesse en l’absence du mari ? | 1= l’attendre  2= aller seule à l’hôpital  3= aller dans ma belle famille  4= aller dans ma famille  5= prévenir voisins/amis  7= Autres 9=nsp | /__/ |
| 704 | Quel est le support spécifique du mari durant la grossesse | 1= rappeler les CPN  2= financer les soins et médicaments  3= aider à respect instructions du médecin  4= aider tâches ménagères  5= accompagner à l’hôpital  7=autre |  |
| 705 | Quelles sortes d’activités ne doit pas faire une femme enceinte ? | | |

706 . Comment qualifiez-vous ces signes/ diagnostic

| N° | **Signes/ diagnostic** | **normal** | **Pas normal** | **Grave/mortel** |
| --- | --- | --- | --- | --- |
| 01 | Hypertension artérielle pendant la grossesse |  |  |  |
| 02 | Douleurs pelviennes pendant la grossesse |  |  |  |
| 03 | Saignement vaginal pendant la grossesse |  |  |  |
| 04 | Saignement vaginal après accouchement |  |  |  |
| 05 | Fièvre (> 24h) après accouchement |  |  |  |
| 06 | Ecoulement vaginal malodorant après accouchement |  |  |  |
| 07 | Travail prolongé de + de 12h |  |  |  |
| 08 | Coups de pieds de l’enfant dans le ventre |  |  |  |
| 09 | Convulsions |  |  |  |
| 10 | Vertiges |  |  |  |
| 11 | Pâleur |  |  |  |
| 12 | Jambes/visage gonflés |  |  |  |
| 13 | Fatigue +++ |  |  |  |
| 14 | Autres signes (à préciser) |  |  |  |

707. Pourquoi avez-vous choisi d’accoucher à l’hôpital (maternité) et non à domicile ?

708. Comment se pratiquait l’accouchement dans votre culture ?

**Vérifier que tout est rempli, REMERCIER L’ENQUETE et arrêter l’interview !**

**Section finale : Enquêtrice, prière de noter :**

1. **les problèmes pour trouver le meilleur répondant**
2. **votre commentaire sur le déroulement de l’interview**
3. votre satisfaction sur la coopération de l’enquêté : 1= TB ; 2= B ; 3= M
